# Comparison of the Neuroprotective and Anti-Inflammatory Effects of the Anthocyanin Metabolites, Protocatechuic Acid and 4-Hydroxybenzoic Acid

**DOI:** 10.1155/2017/6297080

**Published:** 2017-06-27

**Authors:** Aimee N. Winter, Matthew C. Brenner, Noelle Punessen, Michael Snodgrass, Caleb Byars, Yingyot Arora, Daniel A. Linseman

**Affiliations:** ^1^Department of Biological Sciences, University of Denver, Denver, CO 80208, USA; ^2^Eleanor Roosevelt Institute, University of Denver, Denver, CO 80208, USA; ^3^Knoebel Institute for Healthy Aging, University of Denver, Denver, CO 80208, USA

## Abstract

Anthocyanins are being increasingly investigated for their neuroprotective and antineuroinflammatory effects; however, the overall bioavailability of many anthocyanins is relatively low. In contrast, phenolic acids, metabolites of many polyphenols, including anthocyanins, have been shown to accumulate in tissue at higher concentrations than those of parent compounds, suggesting that these metabolites may be the bioactive components of anthocyanin-rich diets. We examined the neuroprotective capacity of two common phenolic acids, 4-hydroxybenzoic acid (HBA) and protocatechuic acid (PCA), in primary cultures of cerebellar granule neurons. Both HBA and PCA are capable of mitigating oxidative stress induced by hydrogen peroxide, which is thought to contribute to neuronal cell death in neurodegeneration. Under conditions of nitrosative stress, which occur during inflammation in the central nervous system, only PCA was neuroprotective, despite similar structural characteristics between HBA and PCA. Intriguingly, this trend was reversed under conditions of excitotoxicity, in which only HBA was neuroprotective. Lastly, we explored the anti-inflammatory activity of these compounds in microglial cells stimulated with lipopolysaccharide. PCA was an effective anti-inflammatory agent, reducing nitric oxide production, while HBA had no effect. These data indicate that phenolic acids possess distinct neuroprotective and anti-inflammatory characteristics that could make them suitable for the treatment of neurodegeneration.

## 1. Introduction

With steady medical advances being made in recent decades, the human population has enjoyed a considerable increase in average life expectancy; however, as the population ages, the incidence of neurodegenerative disease has also increased. Indeed, disorders including Alzheimer's disease, Parkinson's disease, and amyotrophic lateral sclerosis (ALS) have all seen a rise in positive diagnoses, prompting extensive research into the etiology and pathology of these diseases [1]. Despite these efforts, however, the treatments for these diseases remain scarce and are targeted primarily at reducing symptoms rather than at alleviating causes underlying disease pathology and progression.

Neurodegenerative diseases are characterized by the death of specific neuronal populations within the brain, brain stem, and spinal cord, producing significant cognitive and/or motor impairments. Although each disease is uniquely characterized by the type of neurons that are ultimately affected, the underlying causes of neuronal death are thought to be remarkably similar. These include conditions such as oxidative stress, caused by the buildup of reactive oxygen species (ROS) within the cell that damage vital cellular components such as DNA and proteins, nitrosative stress, caused by damaging reactive nitrogen species (RNS) produced in the central nervous system (CNS) under neuroinflammatory conditions, and excitotoxicity, resulting from the overstimulation of neuronal cells by excitatory neurotransmitters, causing massive calcium influx and subsequent activation of cell death signaling cascades [2–6]. The identification of agents targeting multiple aspects of neurodegenerative disease pathology, therefore, provides an appealing therapeutic avenue for treating multiple disorders.

In this regard, polyphenols have emerged as promising therapeutic candidates due to their impressive antioxidant, antineuroinflammatory, and antiapoptotic effects (reviewed by [7]). In particular, anthocyanins, a unique class of flavonoid compounds, show significant potential as a treatment for neurodegenerative disease for these reasons in addition to the observation that they are among the most commonly consumed polyphenolic species around the world [8]. These compounds, responsible for producing the red, blue, and purple pigmentation of many fruits and vegetables, have received significant attention as neuroprotective agents both in vitro and in vivo; however, the efficacy of these compounds for the treatment of neurodegenerative diseases may be limited by their relatively low bioavailability in the brain following ingestion [9–13]. Indeed, anthocyanins are rapidly absorbed and have been shown to accumulate in several areas of the brain; however, the levels at which anthocyanins accumulate are extremely low at only ~0.2 nmol/g of tissue [9–13].

The low levels of circulating anthocyanins are thought to be due in part to extensive metabolism of the parent compounds by gut microflora to form various phenolic acid metabolites and a universal aldehyde metabolite, known as phloroglucinol aldehyde (PGA; 2,4,6-trihydroxy-benzaldehyde) [14–17]. This phenomenon has been described by several studies, and it has been reported that incubation of anthocyanin aglycons with gut microflora results in complete degradation of the parent species to form phenolic acids and aldehydes [17]. Furthermore, circulating concentrations of phenolic acid metabolites derived from anthocyanin degradation such as protocatechuic acid (PCA; 3,4-dihydroxybenzoic acid) have been observed at up to eight times to that of the parent anthocyanins [18]. It has also been reported that PCA remains in relevant tissues longer than its parent anthocyanin compounds [19]. Similarly, gallic acid, another phenolic acid metabolite of some anthocyanin compounds, has also been observed to accumulate in brain tissue at high levels with chronic consumption [20]. Collectively, these observations have led investigators to suggest that phenolic acids and other anthocyanin metabolites are the bioactive components of anthocyanin-rich diets [21]. Studies assessing the neuroprotective and anti-inflammatory effects of these compounds are few in number, however, and have focused predominately on only two phenolic acids, PCA and gallic acid. Thus, the distinct neuroprotective effects of other phenolic acids and aldehydes derived from anthocyanins have not been examined.

We have previously reported that two structurally similar, but distinct anthocyanins, cyanidin-*O*-3-glucoside (kuromanin) and pelargonidin-*O*-3-glucoside (callistephin), display differential neuroprotective effects against a variety of neurotoxic insults. While both callistephin and kuromanin are capable of protecting primary cerebellar granule neurons (CGNs) from mitochondrial oxidative stress and excitotoxicity, only kuromanin is capable of defending neurons from nitrosative stress induced by the nitric oxide donor, sodium nitroprusside (SNP) [22, 23]. In good agreement with these studies, it has also been reported that PCA and gallic acid display differential abilities to interact with lipid-rich environments and prevent lipid peroxidation dependent upon their relative lipophilicity [24]. These studies suggest that different anthocyanins, and likely their respective metabolites, may display distinct neuroprotective effects.

**Figure 1:**
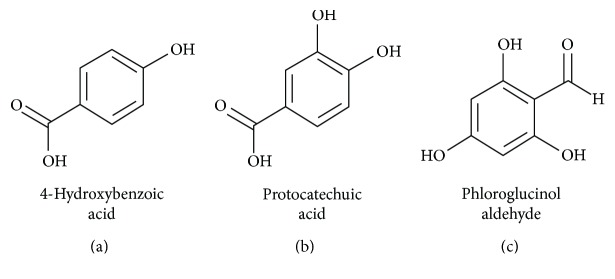
Chemical structures of 4-hydroxybenzoic acid (a), protocatechuic acid (b), and phloroglucinol aldehyde (c).

Following the work of our previous study on the distinct neuroprotective effects of anthocyanins, we examine the differential neuroprotective effects of two phenolic acids, PCA, the primary metabolite of cyanidin-based anthocyanins, such as kuromanin, and 4-hydroxybenzoic acid (HBA), the primary metabolite of pelargonidin-based anthocyanins, such as callistephin. We also explored the neuroprotective effects of the universal anthocyanin metabolite, PGA. While the neuroprotective effects of PCA against a variety of stressors such as hydrogen peroxide and SNP have previously been investigated, the neuroprotective abilities of HBA and PGA have never been explored, and the anti-inflammatory capabilities of these compounds have not yet been defined [25–28]. Moreover, to our knowledge, this is the first study to directly compare the neuroprotective and anti-inflammatory capacities of two distinct but structurally similar phenolic acids as well as PGA (Figure 1) in order to determine their potential efficacy for the treatment of neurodegenerative disease. Our results demonstrate that PCA and HBA display differential neuroprotective and antineuroinflammatory abilities under different neurotoxic conditions, which are described below, while PGA does not display any neuroprotective characteristics.

## 2. Materials and Methods

### 2.1. Materials

Protocatechuic acid was purchased from MP Biomedicals (Solon, OH). Potassium chloride, PGA, 4-hydroxybenzoic acid, glutamic acid, glycine, lipopolysaccharide (LPS) from *E. coli*, bovine serum albumin (BSA), paraformaldehyde, Hoechst 33258, and Tween 20 were purchased from Sigma-Aldrich (St. Louis, MO). Sodium nitroprusside (SNP) was obtained from Calbiochem (San Diego, CA). Basal Medium Eagle's solution, Dulbecco's Modified Eagle's Medium with glucose solution, L-glutamine solution, penicillin/streptomycin solution, and fetal bovine serum (FBS) were purchased from Invitrogen (Grand Island, NY). Nitric oxide assay kits (EMSNO) were obtained from Thermo Scientific (Rockford, IL).

### 2.2. Cell Culture

Cerebellar granule neurons (CGNs) were isolated from 7 day-old Sprague-Dawley rat pups of both sexes (Charles River, Bar Harbor, ME) as previously described [29]. Briefly, cells were plated in poly-L-lysine-coated 6-well plates (35 mm diameter) at a density of approximately 4.0 × 10^6^ cells per well. Cells were maintained in Basal Medium Eagle's supplemented with 2 mM L-glutamine, 25 mM potassium chloride, 10% FBS, and penicillin/streptomycin (100 U/mL/100 *μ*g/mL). After 24 h, CGNs were treated with cytosine arabinoside at a concentration of 10 *μ*M to inhibit the growth of nonneuronal cells. CGN cultures were then maintained in 10% CO_2_ at 37°C for 6-7 days before experiments were performed. This procedure produced cultures that were ~95% pure.

BV2 microglia were maintained in Dulbecco's Modified Eagle's Medium containing 4.5 g/L glucose and supplemented with 10% FBS and penicillin/streptomycin (100 U/mL/100 *μ*g/mL). BV2 microglia were maintained in 5% CO_2_ at 37°C. For experiments, cells were plated in 6-well plates (35 mm diameter) and allowed to reach 80% confluency before treatment.

### 2.3. Treatment of Cell Cultures

#### 2.3.1. CGNs

Culture medium was removed and replaced with medium lacking fetal bovine serum to eliminate the neuroprotective effects of serum that might prevent neuronal apoptosis induced by neurotoxic insult. CGNs were then treated with 250 *μ*M hydrogen peroxide, 100 *μ*M SNP, or 100 *μ*M/10 *μ*M glutamate/glycine alone or in combination with the indicated doses of either PCA or HBA. CGNs were allowed to incubate under these conditions for 24 h prior to assay of neuronal cell death. For all experiments, an untreated control in serum-free medium was used for comparison in assaying cell death.

#### 2.3.2. BV2 Cells

BV2 microglial cells were treated with LPS at a concentration of 1 *μ*g/mL alone or in combination with PCA or HBA at the indicated concentrations in cell culture medium. Cells were allowed to incubate for 24 h prior to collection of culture medium and assay of nitric oxide production.

### 2.4. Assays of Neuronal Cell Death

#### 2.4.1. Nuclear Staining for Apoptosis

Following treatment, CGNs were washed twice with phosphate-buffered saline (PBS, pH = 7.4) and then fixed for 1 h at room temperature with 4% paraformaldehyde. CGNs were then washed again with PBS and stained with Hoechst at a concentration of 10 *μ*g/mL to visualize nuclear morphology. Cells were washed with PBS and imaged using a Zeiss Axiovert-200 M epi-fluorescence microscope. Five bright field and five nuclear images per well were captured to assess cell death with duplicate wells for each treatment composing one experiment. Cells were counted and scored as either viable or apoptotic based on nuclear morphology using images showing decolorized Hoechst fluorescence. CGNs displaying nuclei with fragmented or condensed morphology were scored as apoptotic, with at least 100 cells per treatment per experiment being scored.

#### 2.4.2. MTT Viability Assay

As an alternative means of assessing cell death, some experiments were evaluated using an MTT viability assay. MTT (3-(4,5-dimethylthiazol-2-yl)-2,5-diphenyltetrazolium bromide) is a tetrazolium dye which is reduced by NAD(P)H-dependent cellular oxidoreductase enzymes, primarily within the mitochondria of viable cells, to yield an insoluble formazan derivative which can be solubilized and assayed colorimetrically as an indicator of cell viability. MTT data presented were obtained from duplicate wells per treatment shown for three independent experiments.

### 2.5. Nitric Oxide Assay

Nitric oxide production was assayed in cell culture medium from BV2 cells following treatment using a nitric oxide assay kit (EMSNO) from Thermo Scientific as per the manufacturer's instructions. This kit assays total concentrations of nitrite in solution, one of the major degradation products of nitric oxide, using the Griess method. The concentration of nitrite for each treatment was determined by comparison to a standard curve created using solutions of known nitrite concentrations, with each treatment being performed in duplicate wells.

### 2.6. Statistical Analysis

All experiments in both CGNs and BV2 microglia were performed using duplicate wells with each experiment being performed at least three times. Data are represented as the mean ± standard error of the mean (SEM) for the total number of experiments carried out (*n*). One-way analysis of variance (ANOVA) with a post hoc Tukey's test was used to analyze all data. A *p* value of <0.05 was considered statistically significant.

## 3. Results

### 3.1. Both PCA and HBA Protect CGNs from Oxidative Stress Induced by Hydrogen Peroxide

The role of oxidative stress has been extensively documented in several forms of neurodegenerative disease, and indices of oxidative damage have been observed in tissue from the brains and spinal cords of patients with Alzheimer's disease, Parkinson's disease, and ALS [3, 6]. Hydrogen peroxide (H_2_O_2_) in particular is thought to play a significant role in these diseases, as proteins such as amyloid beta, implicated in the pathogenesis of Alzheimer's disease, and mutant SOD1, associated with familial forms of ALS, are known to mediate neuronal death in part through enhanced production of this toxic species [30, 31]. Therefore, we first evaluated the ability of PCA and HBA to protect CGNs from H_2_O_2_-induced toxicity.

**Figure 2:**
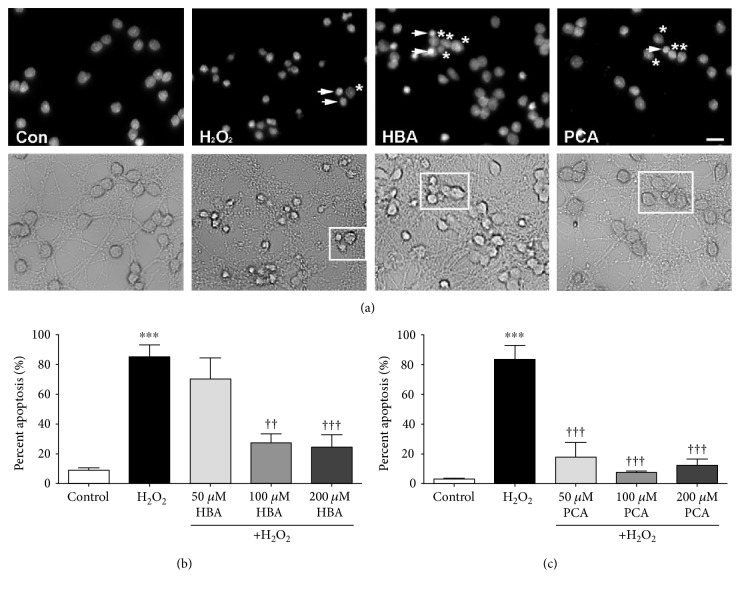
Both HBA and PCA protect CGNs from H_2_O_2_-induced oxidative stress. (a) Representative images of untreated control CGNs (Con), CGNs treated with H_2_O_2_ alone (H_2_O_2_), and CGNs treated in combination with H_2_O_2_ and either 200 *μ*M 4-hydroxybenzoic acid (HBA) or 200 *μ*M protocatechuic acid (PCA). Top panels show decolorized Hoechst fluorescence to visualize nuclei. Bottom panels show bright field images to visualize neuronal cell bodies and processes. Scale bar = 10 *μ*m. Arrowheads indicate apoptotic nuclei, and asterisks indicate healthy nuclei (top panels) of CGNs within the boxes demarcated in the corresponding bottom panels. (b) Quantitative assessment of apoptosis in CGNs treated with H_2_O_2_ alone or in combination with various concentrations of HBA. (c) Quantitative assessment of apoptosis in CGNs treated with H_2_O_2_ alone or in combination with various concentrations of PCA. For quantification, nuclear morphology was assessed, and cells displaying condensed or fragmented nuclei were scored as apoptotic. The percent of all total cells that displayed apoptotic morphology was then determined. Data are represented as mean ± SEM for *n* = 3 experiments. ∗∗∗ indicates *p* < 0.001 in comparison to untreated controls; ††† indicates *p* < 0.001, and †† indicates *p* < 0.01 in comparison to cells treated with H_2_O_2_ alone by one-way ANOVA with a post hoc Tukey's test.

Alone, H_2_O_2_ induced significant levels of cell death, with approximately 80% of CGNs displaying fragmented and/or condensed nuclei as determined with Hoechst staining, which is consistent with apoptosis (Figure 2(a)). Furthermore, H_2_O_2_ treatment caused significant degradation and fragmentation of neuronal processes in addition to shrinkage of neuronal cell bodies, also indicative of cell death. This effect was abrogated by cotreatment of CGNs with either HBA or PCA, which preserved healthy nuclear morphology comparable to that observed in untreated controls as well as preventing the degradation of neuronal processes (Figure 2(a)). Quantification of these data revealed that these protective effects are dose dependent, with higher doses of both HBA and PCA offering greater neuroprotection (Figures 2(b) and 2(c)). Moreover, it was shown that PCA protects neurons more efficiently than HBA, demonstrating significant neuroprotection at lower doses than those required to achieve neuroprotection with HBA.

### 3.2. PCA, but Not HBA, Protects CGNs from Nitrosative Stress Induced by Sodium Nitroprusside

Nitrosative stress is a condition similar to oxidative stress caused by a buildup of toxic RNS such as nitric oxide and peroxynitrite, which are capable of causing oxidative damage to many vital cellular components. S-Nitrosylation of key cellular proteins involved in protein homeostasis and mitochondrial respiration has been described in the context of ALS and Parkinson's disease, respectively, indicating a role for RNS in the underlying pathology of these diseases [32, 33]. Nitric oxide and peroxynitrite are produced in abundance during neuroinflammatory responses in the CNS by activated microglia and reactive astrocytes, which have been shown to play a critical role in the etiology and progression of several neurodegenerative diseases [34]. Thus, compounds that are effectively able to scavenge and detoxify RNS could be of significant therapeutic benefit for the treatment of neurodegeneration. Therefore, we next evaluated the ability of HBA and PCA to mitigate neurotoxicity induced by the nitric oxide donor, SNP.

**Figure 3:**
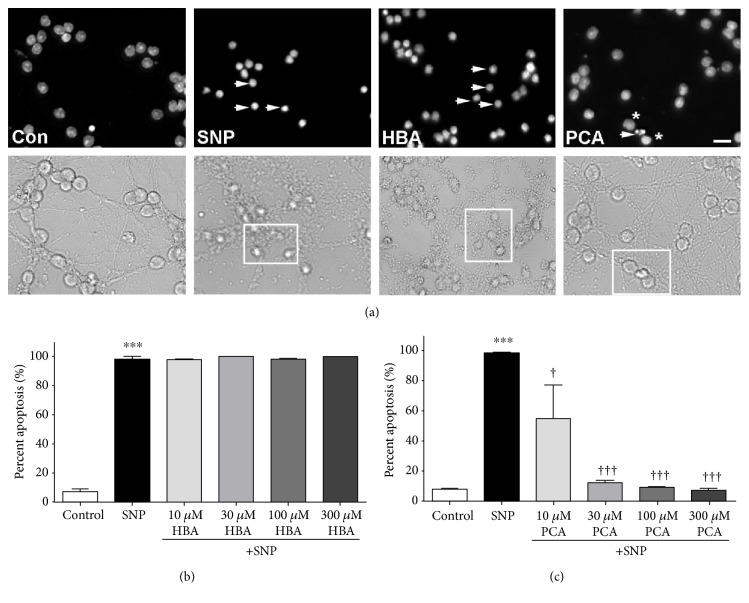
PCA, but not HBA, protects CGNs from nitric oxide-induced toxicity. (a) Representative images of untreated control CGNs (Con), CGNs treated with SNP alone (SNP), and CGNs treated in combination with SNP and either 300 *μ*M 4-hydroxybenzoic acid (HBA) or 300 *μ*M protocatechuic acid (PCA). Top panels show decolorized Hoechst fluorescence to visualize nuclei. Bottom panels show bright field images to visualize neuronal cell bodies and processes. Scale bar = 10 *μ*m. Arrowheads indicate apoptotic nuclei, and asterisks indicate healthy nuclei (top panels) of CGNs within the boxes demarcated in the corresponding bottom panels. (b) Quantitative assessment of apoptosis in CGNs treated with SNP alone or in combination with various concentrations of HBA. (c) Quantitative assessment of apoptosis in CGNs treated with SNP alone or in combination with various concentrations of PCA. For quantification, nuclear morphology was assessed, and cells displaying condensed or fragmented nuclei were scored as apoptotic. The percent of all total cells that displayed apoptotic morphology was then determined. Data are represented as mean ± SEM for *n* = 3 experiments. ∗∗∗ indicates *p* < 0.001 in comparison to untreated controls; ††† indicates *p* < 0.001, and † indicates *p* < 0.05 in comparison to cells treated with SNP alone by one-way ANOVA with a post hoc Tukey's test.

**Figure 4:**
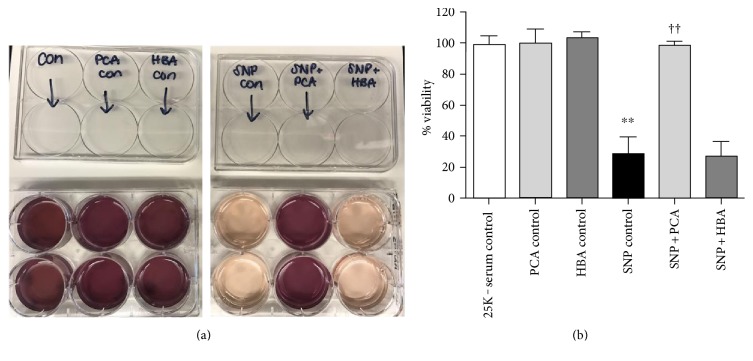
MTT viability assay of SNP-treated CGN cultures. (a) Representative MTT images of untreated control CGNs, CGNs treated with 100 *μ*M protocatechuic acid (PCA) or 100 *μ*M 4-hydroxybenzoic acid (HBA) alone, CGNs treated with sodium nitroprusside alone (SNP), and CGNs treated in combination with SNP and either PCA or HBA. (b) Quantitative assessment of cell viability. Data are represented as mean ± SEM for *n* = 3 experiments, each performed in duplicate. ∗∗ indicates *p* < 0.01 in comparison to untreated control, and †† indicates *p* < 0.01 in comparison to cells treated with SNP alone, by one-way ANOVA with a post hoc Tukey's test.

Treatment of CGNs with SNP alone produced substantial neuronal death, causing extensive nuclear condensation and almost complete obliteration of neuronal processes (Figure 3(a)). Cotreatment with HBA was unable to attenuate this effect with these cells displaying nuclear morphology and fragmented processes similar to that of cells treated with SNP alone (Figure 3(a)). In striking contrast to these results, PCA offered complete protection from this insult and preserved healthy nuclear morphology and neuronal processes (Figure 3(a)). Even at doses as low as 10 *μ*M, cotreatment with PCA provided significant neuroprotection from nitric oxide-induced death, and this effect increased with PCA concentration, while HBA was unable to defend neurons from this insult at any of the doses tested (Figures 3(b) and 3(c)). In agreement with the results obtained utilizing nuclear morphology and neurite fragmentation as morphologic indices of cell death, quantitative assessment of cell viability using an MTT assay revealed the same conclusion; PCA essentially completely protected CGNs from SNP-induced neurotoxicity while HBA had no significant neuroprotective effect under these conditions (Figures 4(a) and 4(b)).

### 3.3. HBA, but Not PCA, Protects CGNs from Glutamate-Induced Excitotoxicity

Excitotoxicity is a process specific to neurons in which overstimulation by excitatory neurotransmitters, such as glutamate, causes massive calcium influx from the extracellular space, triggering the activation of a number of cell death cascades, such as calpain-dependent apoptosis [5]. Additionally, disturbances in calcium homeostasis can cause membrane depolarization in mitochondria, resulting in enhanced production of ROS, and release of apoptogenic factors. Considerable evidence for the involvement of excitotoxicity in Alzheimer's disease, Parkinson's disease, and ALS has accumulated and suggests that ameliorating excitotoxic effects could be a viable therapeutic approach to treating these diseases [35–38]. Thus, we examined the capacity of HBA and PCA to defend neurons from excitotoxic conditions induced by stimulation with the excitatory neurotransmitter, glutamate.

**Figure 5:**
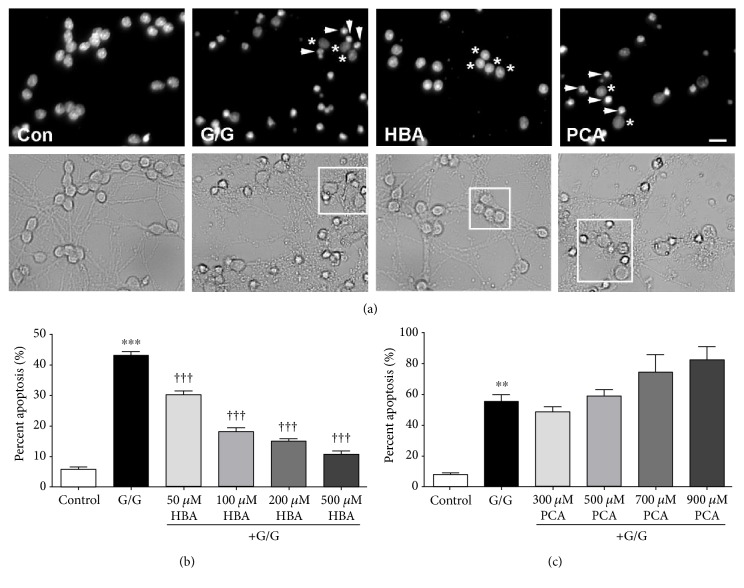
HBA, but not PCA, protects CGNs from glutamate-induced excitotoxicity. (a) Representative images of untreated control CGNs (Con), CGNs treated with glutamate alone (G/G), and CGNs treated in combination with glutamate and either 500 *μ*M 4-hydroxybenzoic acid (HBA) or 500 *μ*M protocatechuic acid (PCA). Top panels show decolorized Hoechst fluorescence to visualize nuclei. Bottom panels show bright field images to visualize neuronal cell bodies and processes. Scale bar = 10 *μ*m. Arrowheads indicate apoptotic nuclei, and asterisks indicate healthy nuclei (top panels) of CGNs within the boxes demarcated in the corresponding bottom panels. (b) Quantitative assessment of apoptosis in CGNs treated with glutamate alone or in combination with various concentrations of HBA. (c) Quantitative assessment of apoptosis in CGNs treated with glutamate alone or in combination with various concentrations of PCA. For quantification, nuclear morphology was assessed, and cells displaying condensed or fragmented nuclei were scored as apoptotic. The percent of all total cells that displayed apoptotic morphology was then determined. Data are represented as mean ± SEM for *n* = 3 experiments. ∗∗∗ indicates *p* < 0.001 and ∗∗ indicates *p* < 0.01 in comparison to untreated controls; ††† indicates *p* < 0.001 in comparison to cells treated with glutamate alone by one-way ANOVA with a post hoc Tukey's test.

When treated with glutamate alone, CGNs experienced approximately 50% cell death evidenced by nuclear condensation and fragmentation as well as moderate degradation of neuronal processes (Figure 5(a)). HBA significantly protected CGNs from glutamate-induced excitotoxicity, preserving nuclear morphology and neuronal processes (Figure 5(a)). As with H_2_O_2_, this effect was dose dependent, with the greatest protection occurring with the highest dose of HBA examined in this experiment in direct contrast to PCA (Figure 5(b)). Under these conditions, PCA displayed no neuroprotective effects, actually displaying a trend towards enhancing neuronal death induced by glutamate excitotoxicity at higher concentrations, although this trend did not reach statistical significance (Figure 5(c)).

### 3.4. PGA, a Universal Anthocyanin Metabolite, Does Not Protect CGNs from Oxidative Stress, Nitrosative Stress, or Excitotoxicity

Metabolism of anthocyanins has been shown to produce both phenolic acids, which retain unique chemical structures derived from the parent compounds, and a universal aldehyde metabolite, known as phloroglucinol aldehyde [14–17]. Since metabolism of anthocyanins by gut microflora results in the production of both phenolic acids and aldehydes, it is possible that some of the beneficial health effects observed with anthocyanin-rich diets could be due to the activity of both phenolic acids and PGA. While the neuroprotective capabilities of some phenolic acids, such as PCA, have been explored, the neuroprotective capabilities of PGA have not been previously examined. Therefore, we next assessed the ability of PGA to defend CGNs from various stressors.

**Figure 6:**
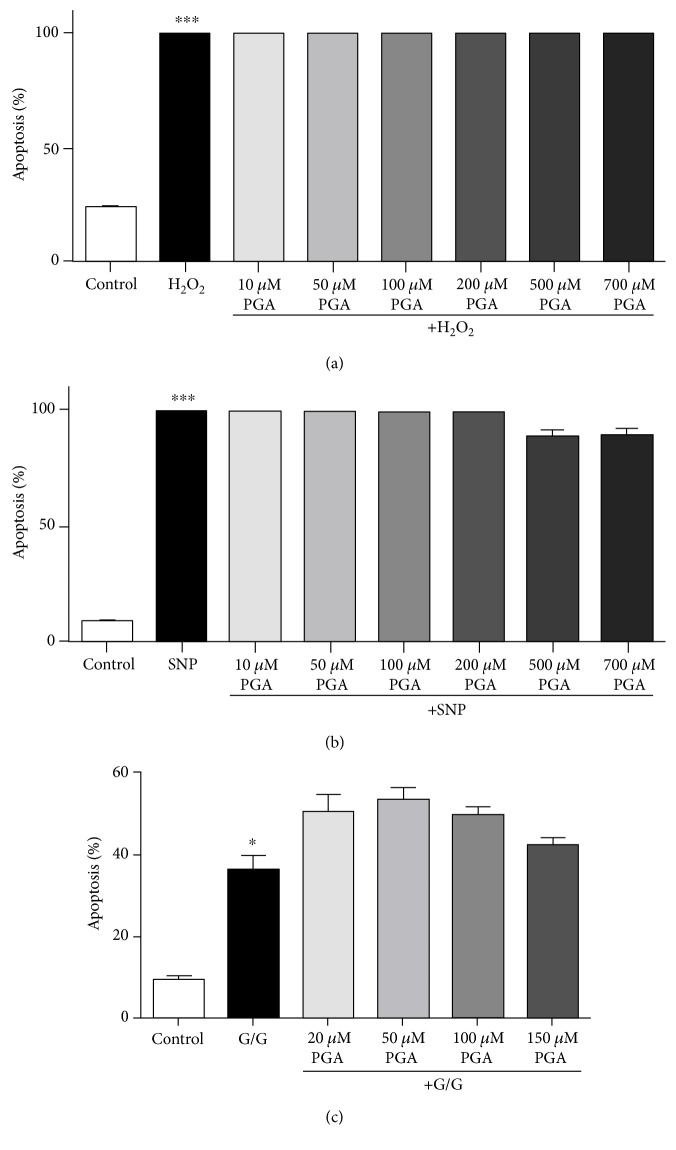
PGA does not protect CGNs from death induced by various neuronal stressors. (a) Quantitative assessment of apoptosis in CGNs treated with hydrogen peroxide alone or in combination with various concentrations of PGA. (b) Quantitative assessment of apoptosis in CGNs treated with SNP alone or in combination with various concentrations of PGA. (c) Quantitative assessment of apoptosis in CGNs treated with glutamate alone or in combination with various concentrations of PGA. For quantification, nuclear morphology was assessed, and cells displaying condensed or fragmented nuclei were scored as apoptotic. The percent of all total cells that displayed apoptotic morphology was then determined. Data are represented as mean ± SEM for *n* = 3 experiments. ∗∗∗ indicates *p* < 0.001, and ∗ indicates *p* < 0.01 in comparison to untreated controls by one-way ANOVA with a post hoc Tukey's test. There are no significant differences between cells treated with PGA and cells treated with insults alone.

We first explored the ability of PGA to protect CGNs from oxidative stress induced by H_2_O_2_. Treatment with H_2_O_2_ alone significantly increased levels of cellular apoptosis; however, cotreatment of cells with PGA was unable to protect CGNs from this insult at any of the doses we examined (Figure 6(a)). Next, we evaluated the neuroprotective capacity of PGA in the context of nitric oxide toxicity induced by SNP. CGNs treated with SNP alone displayed a dramatic increase in levels of apoptosis, and cotreatment with PGA did not reduce apoptosis in comparison to cells treated with SNP alone (Figure 6(b)). Lastly, we assessed the neuroprotective effects of PGA against glutamate excitotoxicity. Cells treated with glutamate alone showed a significant increase in the number of apoptotic cells in comparison to untreated controls (Figure 6(c)). Cotreatment with PGA did not attenuate glutamate-induced cell death (Figure 6(c)).

### 3.5. PCA, but Not HBA, Attenuates Lipopolysaccharide-Induced Microglial Inflammation in the BV2 Cell Line

Microglia are the primary mediators of inflammatory immune responses in the CNS. While beneficial in the short term for neuronal protection and repair from foreign invaders, prolonged inflammation of these cells is neurotoxic and has been implicated as a major contributor to the neuronal death underlying neurodegenerative disease [39]. Indices of microglial inflammation, such as enhanced production of inflammatory cytokines, induction of proinflammatory proteins such as COX-2 and inducible nitric oxide synthase (iNOS), and microglial proliferation have been described in Alzheimer's disease, Parkinson's disease, and ALS [34, 40–45]. Collectively, these studies demonstrate that microglial inflammation is a significant feature of these diseases, making this facet of the neurodegenerative process an appealing therapeutic target. One of the many hallmarks of microglial inflammation is the upregulation of iNOS and subsequent production of large quantities of nitric oxide [46]. Therefore, we assessed the ability of HBA and PCA to alter nitric oxide production in the BV2 microglial cell line following treatment with LPS.

**Figure 7:**
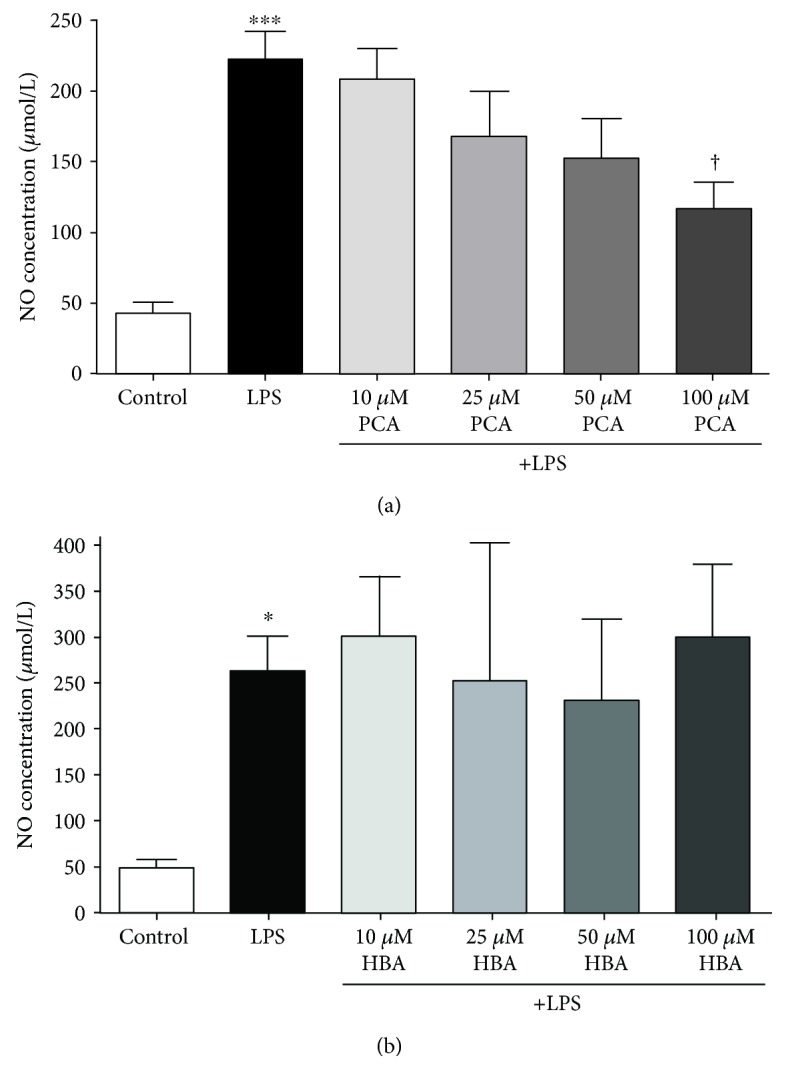
PCA, but not HBA, attenuates inflammation induced by LPS in BV2 microglia. (a) Quantitative assessment of nitric oxide production in untreated, BV2 microglia, and microglia stimulated with LPS alone or in combination with various concentrations of PCA. Nitric oxide production was determined using the Griess method to measure nitrite, a major degradation product of nitric oxide, in cell culture medium incubated with BV2 microglia. (b) Quantitative assessment of nitric oxide production by BV2 microglia stimulated with LPS alone or in combination with various concentrations of HBA. Nitric oxide production was quantified as in (a). Data are represented as mean ± SEM for *n* = 3 experiments for nitric oxide assay. ∗∗∗ indicates *p* < 0.001, and ∗ indicates *p* < 0.05 in comparison to untreated controls; † indicates *p* < 0.05 in comparison to cells treated with LPS alone by one-way ANOVA with a post hoc Tukey's test.

LPS induced a significant inflammatory response in BV2 microglia marked by a considerable increase in nitric oxide production (Figures 7(a) and 7(b)). Cotreatment of BV2 cells with both LPS and PCA dose dependently reduced nitric oxide production by a significant amount (Figure 7(a)). Cotreatment with HBA and LPS, however, did not produce a significant effect on microglial inflammation, with nitric oxide production remaining largely unchanged in comparison to microglia treated with LPS alone (Figure 7(b)).

## 4. Discussion

Diets rich in anthocyanins and other polyphenols are associated with a myriad of health benefits, including decreased risk of developing cancer, cardiovascular disease, and neurodegenerative disease, particularly Parkinson's disease [47, 48]. As anthocyanins are known to mitigate multiple facets of the neurodegenerative process thought to contribute to neuronal cell death (reviewed by [49]), this finding is perhaps unsurprising; however, current research suggests that it is likely anthocyanin metabolites produced after anthocyanin ingestion are truly responsible for mediating these positive effects in vivo. We have previously compared the neuroprotective abilities of two anthocyanin species, callistephin and kuromanin, against mitochondrial oxidative stress, nitrosative stress, and excitotoxicity and found that slight structural variances in anthocyanin structure significantly influence the neuroprotective capacity of these compounds against different neurotoxic insults [22, 23]. Given these data in conjunction with the observation that phenolic acid metabolites from anthocyanins are likely responsible for mediating the beneficial effects of these compounds in vivo, it is equally important to determine if different anthocyanin metabolites display differential neuroprotective functions that may influence their overall effectiveness as potential therapeutic agents in neurodegenerative disease. However, while the neuroprotective capacity of some anthocyanin metabolites has been previously evaluated, a systemic comparison of the effects of metabolites from anthocyanins against a broad range of neurotoxic insults has never been conducted. Here, we evaluated the broad neuroprotective and anti-inflammatory effects of two phenolic acid metabolites, HBA and PCA, which are derived from pelargonidin-based and cyanidin-based anthocyanins, respectively, and the universal aldehyde metabolite, PGA.

Our results demonstrate that HBA and PCA display both similar and distinct neuroprotective effects against several neurotoxic stressors. While both compounds are capable of defending CGNs from H_2_O_2_-induced oxidative stress, consistent with previous studies conducted with PCA [25–28], their protective effects diverge in the context of nitrosative stress and glutamate excitotoxicity. In agreement with prior reports, PCA is a highly effective neuroprotective agent against nitric oxide-induced death following treatment with SNP [25]. These results are in remarkable contrast to those observed for HBA, which demonstrate that this compound is incapable of mitigating nitric oxide toxicity to any degree. Our previous work with kuromanin and callistephin, parent compounds of PCA and HBA, respectively, also displayed this trend, with kuromanin protecting CGNs to a significant degree from SNP toxicity, while the closely related anthocyanin, callistephin, showed no effect on nitric oxide-induced death [23]. This previous work also demonstrated that protection from nitric oxide by kuromanin appeared to be catechol-dependent, a structural feature that both kuromanin and its phenolic acid metabolite, PCA, share, but which is notably lacking from the structure of both callistephin and its metabolite, HBA (Figure 1). Thus, it is very likely that this slight structural difference between HBA and PCA that gives PCA a catechol moiety is responsible for their vastly different neuroprotective abilities against nitric oxide toxicity.

This trend appears to be reversed in the context of excitotoxicity. In CGNs stimulated with glutamate, HBA offered significant neuroprotection, while PCA treatment had no effect on neuronal viability against this insult. This result was unexpected as we have previously shown that both callistephin and kuromanin, two of the parent compounds of these phenolic acids, are potent inhibitors of excitotoxic cell death in CGNs [23]. Furthermore, it has been reported that PCA is capable of reducing increases in intracellular calcium concentrations in primary cortical neurons treated with amyloid beta, which are thought to occur through activation of *N*-methyl-*D*-aspartate (NMDA) receptors at the cell surface [50, 51]. As massive calcium influx through NMDA receptors is also a major feature of glutamate excitotoxicity, it is somewhat surprising that PCA does not protect CGNs from glutamate treatment. The possibility remains, however, that the protective effects of PCA on neuronal viability in the context of amyloid beta toxicity are due to mechanisms other than its ability to attenuate disruptions in calcium homeostasis. These mechanisms could be distinct from those observed under excitotoxic conditions, which could render PCA ineffective for alleviating excitotoxic stress. Alternatively, this could also suggest that the ability of PCA to regulate calcium homeostasis is not sufficient to protect CGNs from glutamate excitotoxicity. This then suggests two possible mechanisms to explain the ability of the closely related HBA to preserve neuronal viability following excitotoxic insult in contrast to PCA. One simple explanation for the differences observed between these compounds is that they may both regulate calcium homeostasis, but HBA may do so more effectively than PCA, which could in turn prevent activation of downstream proapoptotic signaling to a greater extent. Conversely, preservation of neuronal viability by HBA but not PCA could be reliant on the ability of HBA to activate prosurvival or inhibit proapoptotic signaling pathways involved in excitotoxic death, an ability that PCA may lack due to structural differences between these compounds. However, since neither PCA nor HBA has been examined for their neuroprotective effects against excitotoxicity until now, further exploration of this topic is needed to define the neuroprotective mechanism of HBA treatment under these conditions.

PGA was also evaluated for its neuroprotective capabilities in the context of both oxidative and nitrosative stress and glutamate excitotoxicity. Our data revealed that PGA is a poor neuroprotective agent as this compound was unable to mitigate toxicity induced by hydrogen peroxide, SNP, and glutamate treatment. This was somewhat surprising as recent research using a luciferase reporter assay in CHO cells suggests that PGA is a potent inducer of Nrf2 expression, whereas PCA and cyanidin-3-*O*-glucoside did not appear to alter Nrf2 expression [52]. Moreover, this study revealed that metabolism of anthocyanins by gut microflora appears to be necessary to observe changes in the expression of Nrf2 effector genes, such as quinone oxidoreductase 1, suggesting that anthocyanin metabolites, particularly PGA, and not anthocyanins themselves, may be responsible for Nrf2 upregulation [52]. Given this finding, it is somewhat surprising that PGA did not protect CGNs from oxidative stress; however, this could be due to the fact that primary neurons have a relatively weak Nrf2 response in comparison to other cells in the brain such as astrocytes [53]. Thus, while PGA does not appear to be directly neuroprotective, it is possible that this compound could mediate neuroprotective effects in vivo through regulation of Nrf2 and its effectors in surrounding glial cells, which may enhance the neuroprotective phenotype of these cells, or prevent glial inflammation in the context of neurodegeneration. Further study is needed to test this hypothesis.

Finally, we assessed the ability of HBA and PCA to attenuate LPS-induced inflammation in the BV2 microglial cell line. Under inflammatory conditions, we again observed a stark difference between the abilities of HBA and PCA, with PCA acting in an anti-inflammatory capacity, while HBA treatment produced no effect on microglial inflammation. This was assessed by the ability of these compounds to reduce nitric oxide production, a hallmark of microglia-mediated inflammation in the CNS [46]. PCA effectively reduced nitric oxide production; however, HBA had no effect on nitric oxide levels. Intriguingly, another anthocyanin metabolite, gallic acid, has also been shown to reduce microglial inflammation in a similar manner, although the effects of PCA and HBA in BV2 microglia have not been evaluated until now. Reductions in inflammation by gallic acid were reported to prevent microglial-mediated toxicity in cocultured neurons, suggesting that some anthocyanin metabolites may mediate neuroprotective effects in vivo through amelioration of microglial inflammation [54]. Despite these observations, the mechanism underlying PCA's ability to attenuate neuroinflammatory responses in microglia is unknown; however, reductions in nitric oxide production suggest that PCA may modulate expression of proteins, such as iNOS, or regulators of the genes encoding proinflammatory mediators, such as nuclear factor-κB. This phenomenon has been well established in BV2 microglia treated with extracts rich in anthocyanins, which may share this ability with some phenolic acid metabolites. A lack of protection by HBA also suggests this mechanism as differences in structural features between HBA and PCA may allow them to regulate different signaling pathways, as suggested above. Nevertheless, further study is needed to confirm or refute this hypothesis.

## 5. Conclusions

Collectively, these data highlight the intriguing neuroprotective and anti-inflammatory differences in anthocyanin metabolites, while also indicating some limitations of these compounds to mitigate various factors involved in the neurodegenerative process. For example, while both HBA and PCA may effectively target toxicity induced by ROS, only PCA targets microglial inflammation and nitrosative stress, and only HBA targets excitotoxicity. However, the ideal therapeutic candidate for the treatment of neurodegeneration would target all four of these aspects of disease. It is interesting to note, then, that PCA and HBA display complimentary effects, suggesting that combination treatment with both of these agents may be an effective therapeutic strategy to target a broader range of factors involved in the neurodegenerative process, which could produce greater therapeutic effects in preclinical models of neurodegenerative disease than administration of either compound alone. This is a particularly appealing strategy in light of several recent in vivo studies demonstrating that supplementation with PCA alone is an effective therapeutic treatment in mouse models of Parkinson's disease, Alzheimer's disease, and D-galactose-induced accelerated aging [55–57]. Also, it is important to note that although they are discussed here in the context of anthocyanin metabolism, both HBA and PCA can be produced by the metabolism of other polyphenolic species. As the neuroprotective and anti-inflammatory effects of these compounds appear to be dose dependent, identifying parent compounds that can be metabolized to produce greater concentrations of PCA and HBA than anthocyanins in vivo could be an effective strategy for identifying new therapeutic candidates for testing in preclinical models of disease. Finally, it should be noted that although PCA and HBA display direct neuroprotective effects in vitro, the concentrations necessary to achieve these effects are quite high, typically in the 10–100 *μ*M range. These concentrations are not observed in vivo and therefore, the benefits of an anthocyanin-rich diet on CNS health are likely due to a combinatorial effect of multiple antioxidant and anti-inflammatory compounds. Nonetheless, it is important to establish the relative neuroprotective and anti-inflammatory properties of individual metabolites as a starting point for preclinical testing and possible therapeutic development.

In summary, both HBA and PCA display distinct neuroprotective effects in vitro in primary CGNs, suggesting that these compounds warrant further exploration both alone and in combination to further define their neuroprotective and anti-inflammatory mechanisms under diverse stress conditions. Moreover, further exploration of these compounds in preclinical models of disease is warranted as both compounds target multiple aspects of neurodegenerative disease pathology. In particular, the use of HBA and PCA in combination could be of great therapeutic potential owing to the diverse and complimentary neuroprotective effects of these compounds against oxidative stress, nitrosative stress, and excitotoxicity as well as the antineuroinflammatory effects of PCA [58].
